# Post-operative Saddle Pulmonary Embolism: A Case Report

**DOI:** 10.7759/cureus.69175

**Published:** 2024-09-11

**Authors:** Aaditya Jandhyala, Jasra Elahi, Latha Ganti, Kevin M Sherin

**Affiliations:** 1 Biomedical Sciences, University of Central Florida, Orlando, USA; 2 Biotechnology, Rutgers University, New Jersey, USA; 3 Emergency Medicine and Neurology, University of Central Florida, Orlando, USA; 4 Research, Orlando College of Osteopathic Medicine, Winter Garden, USA; 5 Medical Science, The Warren Alpert Medical School of Brown University, Providence, USA; 6 Primary Care, Orlando College of Osteopathic Medicine, Orlando, USA

**Keywords:** deep vein thrombosis (dvt), emergency medicine, lower-extremity surgery, post-op complications, saddle pulmonary embolism

## Abstract

Pulmonary embolisms are serious complications that can arise from surgical procedures involving the extremities due to the risk of deep vein thrombosis (DVT) and subsequent embolism. This life-threatening condition occurs when an embolus lodges at the bifurcation of the main pulmonary arteries, compromising blood flow to the lungs. Treatment options for pulmonary embolism primarily include anticoagulation therapy, thrombolysis, thrombectomy, and inferior vena cava (IVC) filter placement. Given that cases of saddle pulmonary embolism are rare but potentially fatal, healthcare providers must maintain a high index of suspicion and implement rigorous preventive measures to mitigate the risk in surgical patients.

## Introduction

A post-operative pulmonary embolism (PE) is a severe and potentially fatal complication that can occur after surgical procedures, particularly those involving the extremities. This condition arises when a blood clot, typically from a deep vein thrombosis (DVT) in the legs, travels through the bloodstream and lodges in the pulmonary arteries, obstructing blood flow to the lungs [1]. PEs are a significant cause of morbidity and mortality in surgical patients and pose a substantial challenge to healthcare providers because they are the third most common cause of cardiovascular-related fatality [2].

Patients who develop PEs present with signs and symptoms such as sudden shortness of breath, chest pain, tachycardia, and sometimes even hemoptysis. The clinical presentation of a PE can vary widely, which makes diagnosis challenging [3]. Risk factors for a post-operative PE include prolonged immobility, major surgery, advanced age, obesity, a history of thromboembolic events, and certain inherited or acquired hypercoagulable states [4].

Despite advancements in surgical techniques and post-operative care, the incidence of PEs remains a concern. Preventive measures, such as anticoagulant prophylaxis and mechanical compression devices, are critical in mitigating the risk of PEs [5]. However, even with these preventive strategies, cases of patients suffering from PEs still occur, underscoring the need for vigilance and prompt intervention.

Current treatment options for PEs include anticoagulation therapy, which is the mainstay of treatment, and the placement of an inferior vena cava (IVC) filter in patients who have contraindications to anticoagulation or in those who experience recurrent embolism despite adequate anticoagulation [6]. Thrombolytic therapy and surgical embolectomy may be considered in severe cases where rapid resolution of the embolism is necessary to prevent hemodynamic collapse [7,8].

Given the potentially devastating consequences of a post-operative PE, ongoing research and improvements in preventive and therapeutic strategies are essential to enhance patient outcomes and reduce the incidence of this life-threatening complication [9].

## Case presentation

A 74-year-old man with a history of hypertension and coronary artery disease presented to the emergency department (ED) with right-sided chest pain described as "gas pain." Four days prior, the patient had been discharged after having undergone rotator cuff surgery under general anesthesia. The patient had completed a stress test one month prior to surgery and was deemed medically stable by his cardiologist. He reported occasional shortness of breath, which he attributed to physical therapy. The patient had stopped his daily aspirin regimen before the surgery and had not yet restarted it. During the ED visit, aspirin was administered.

The patient was awake, alert, and in no acute distress on physical examination. His vital signs were: blood pressure 173/95 mmHg, pulse 75 beats per minute, respiratory rate 18 breaths per minute, temperature 98.4°F, and oxygen saturation 95% on room air. His physical examination was unremarkable, with bilateral normal heart and breath sounds.

**Figure 1:**
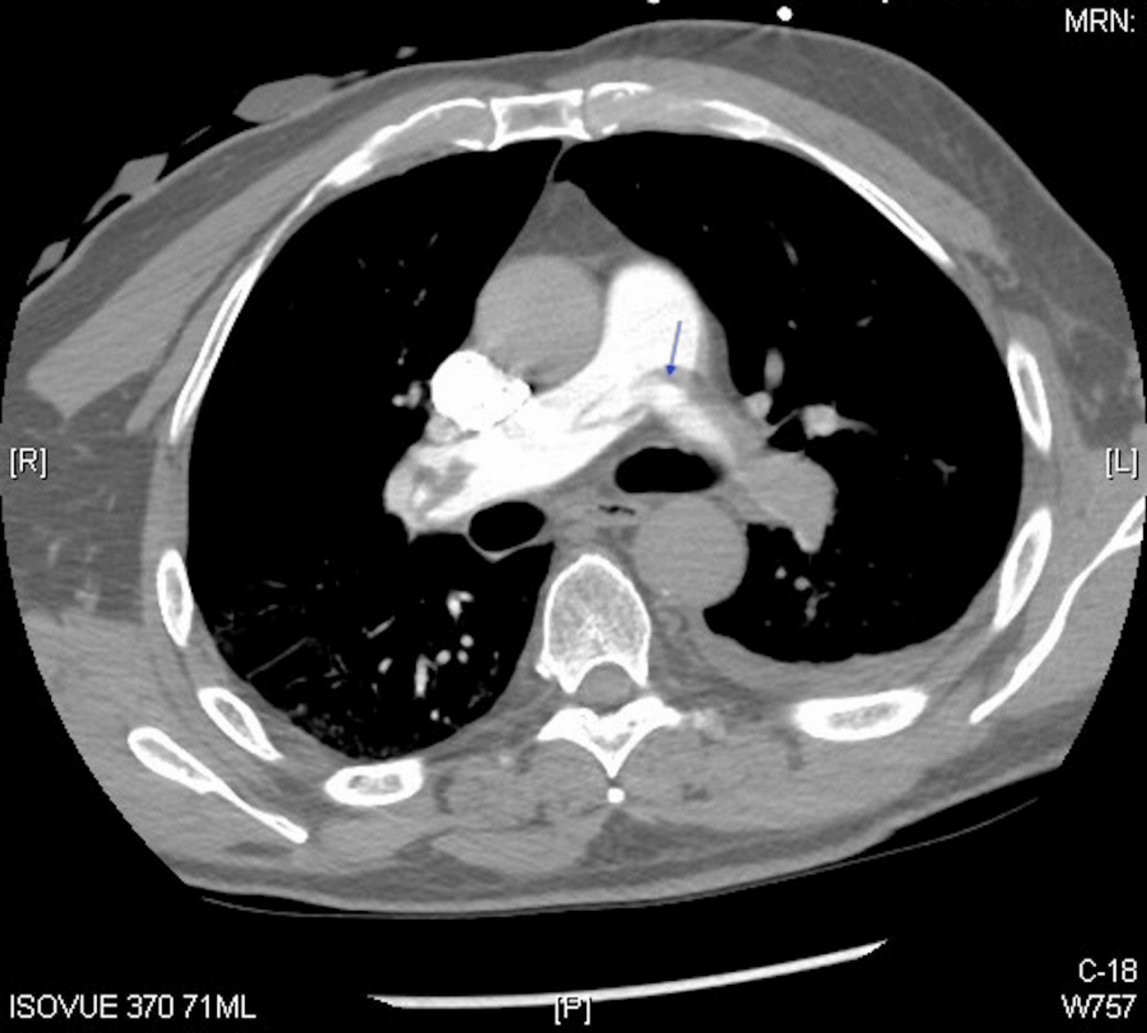
Computed tomography angiography (CTA) demonstrating a saddle pulmonary embolus (arrow)

A chest X-ray revealed a mild deviation of the trachea to the right, possibly due to an underlying mass, but no acute process was evident. Pulmonary embolism was the top differential diagnosis, so a computed tomography angiography (CTA) of the chest was performed and revealed a significant burden of acute pulmonary embolism bilaterally, more significant on the left side, including a saddle embolus. There was no evidence of cardiac strain by CT criteria. The CTA also noted subtle peripheral areas of airspace consolidation in the left lower lobe, consistent with peripheral lung infarcts and a small left pleural effusion. An incidental finding of a considerable heterogeneous nodular enlargement of the left lobe of the thyroid gland was also noted (Figure 1).

Given the patient's stability and the absence of acute right heart strain, the consulting radiologist deemed immediate intervention unnecessary. Laboratory tests revealed an elevated troponin level, leading to a secondary impression of ruling out acute coronary syndrome (Table 1).

**Table 1:** Patient's laboratory results GFR: glomerular filtration rate

Test	Value	Normal range and units
Sodium	136	135 - 145 mmol/L
Potassium	4.1	3.5 - 5.3 mmol/L
Chloride	100	99 - 111 mmol/L
Carbon dioxide	26	21 - 32 mmol/L
Blood urea nitrogen	38 (High)	7 - 22 mg/dL
Creatinine	1.4 (High)	0.6 - 1.3 mg/dL
Estimated GFR	52	> 60
Glucose	101	70 - 110 mg/dL
Calcium	9.4	8.4 - 10.2 mg/dL
Troponin	1.05 (High)	0.0-0.4 ng/mL
D-dimer	1200 (High)	< 500 ng/mL
White blood cell count	14.5 (High)	4.1 - 9.3 K/mm3
Hemoglobin	14.0	13.8 - 17.2 gm/dL
Hematocrit	42.1	40.6 - 51.8 %
Platelet count	317	150 - 450 K/mm3

It should be noted that although the D-dimer was done and was elevated, it would not have changed management in this case, as the pre-test probability of a PE would have been too high to forego a CTA chest. Although the elevated troponin was likely due to heart strain, the troponins were trended.

The transthoracic echocardiogram performed by cardiology showed an average left ventricle cavity size with mildly increased wall thickness. Systolic function was at the lower limits of normal, with an estimated ejection fraction of 45-50%. There was Grade II diastolic dysfunction and mild regurgitation of the mitral valve. The tricuspid valve showed moderate regurgitation, and the right atrium was mildly dilated. There was a septal aneurysm and moderate elevation of pulmonary artery pressures.

Venous duplex ultrasonography revealed bilateral lower extremity DVTs. The patient was admitted to the medicine service for the placement of an IVC filter, due to the significant clot burden. In addition to the IVC filter, the patient was started on anticoagulation therapy with apixaban. He was discharged on hospital day five with instructions for outpatient follow-up with his primary care physician and a hematologist.

## Discussion

The development of a saddle PE in this patient underscores the significant risk of venous thromboembolism (VTE) following surgery, even in the presence of preventive measures [7,8]. Notably, the discovery of a PE via chest CTA, despite the absence of classic signs and symptoms, highlights the importance of maintaining a high index of suspicion for venous thromboembolism (VTE) in post-operative patients. Virchow's triad-venous stasis, endothelial injury, and hypercoagulability provide a framework for understanding the etiology of VTE [9]. In addition to trauma which can be minor [10], pulmonary emboli can also arise from other pathologies comprising Virchow’s triad. These include hypercoagulability as seen in COVID-19 for example [11,12].

Surgical interventions, especially those involving major orthopedic procedures like rotator cuff surgery, significantly elevate the risk of VTE due to prolonged immobility, direct vascular injury, and the hypercoagulable state [7]. In this case, the patient had undergone rotator cuff surgery and had multiple risk factors for VTE, including advanced age and a history of coronary artery disease. However, he was not obese, did not smoke, and maintained an active lifestyle, which are typically protective factors [8].

The absence of immediate postoperative anticoagulation and the discontinuation of daily aspirin therapy likely contributed to the development of a PE [7]. This case highlights the critical need for vigilant post-operative monitoring and timely intervention. The management of the patient's PE included the placement of an IVC filter to prevent further emboli and the initiation of anticoagulation therapy [8,9]. 

**Figure 2:**
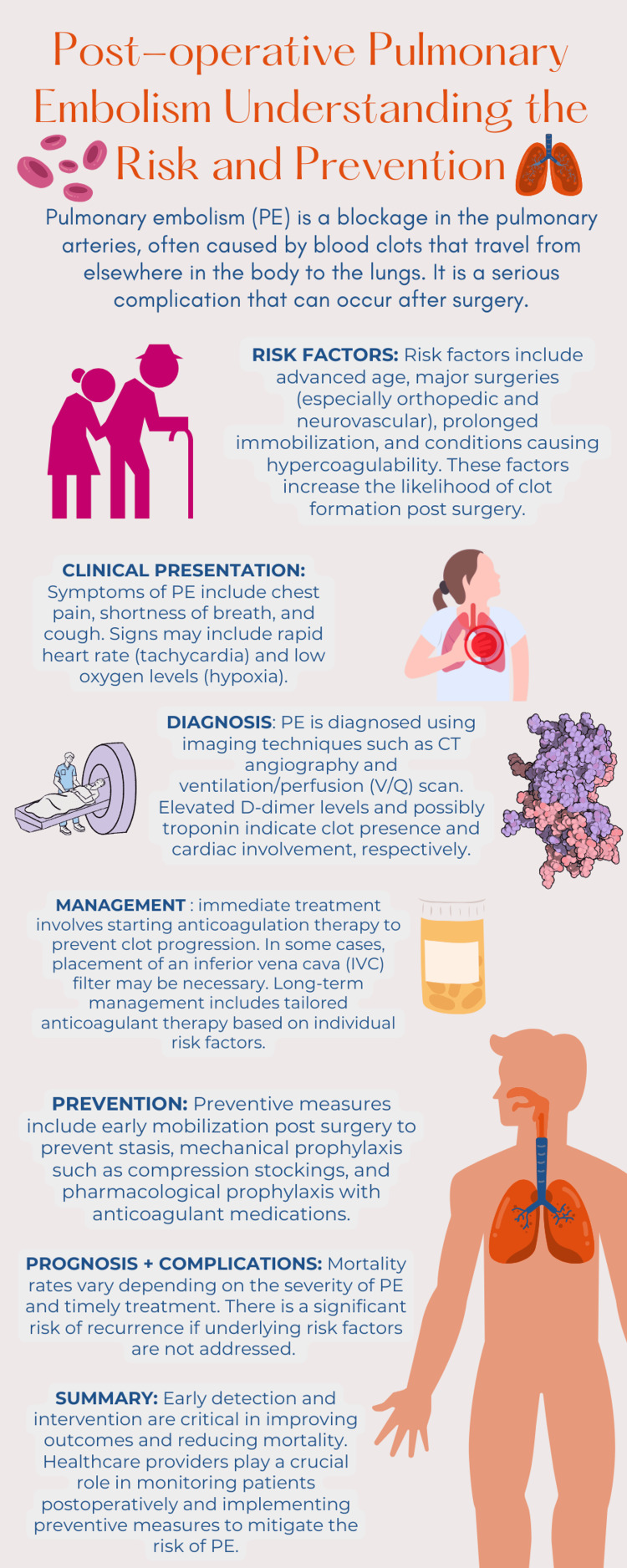
Infographic summarizing ways to mitigate the risk of pulmonary embolism. Image Credit: Author Jasra Elahi via canva.com

Post-operative patients, particularly those undergoing major surgeries, should be closely monitored for signs of VTE [7]. Prophylactic measures, including early mobilization and appropriate anticoagulation, are essential to mitigate this risk (Figure 2).

Early detection and intervention can significantly improve prognosis and reduce the likelihood of severe complications such as saddle PEs. This case reinforces the necessity of a comprehensive approach to VTE prevention and management in the post-operative setting [8,9].

## Conclusions

Given the patient's recent rotator cuff surgery and otherwise limited risk factors, the development of a saddle PE was unexpected. This case underscores the heightened risk of thromboembolic events in post-operative patients, emphasizing the critical need for vigilance in monitoring and managing such complications. Age-related vascular changes and the hypercoagulable state induced by surgery likely contributed to the formation of a PE despite the absence of traditional risk factors like obesity or smoking.

Clinicians should maintain a high index of suspicion for PEs in post-operative patients, especially those exhibiting cardiopulmonary signs and symptoms, even when classic risk factors are absent. Early recognition and intervention are paramount to optimizing outcomes and reducing the risk of recurrence in these vulnerable patient populations.

## References

[1] Goldhaber SZ, Bounameaux H (2012). Pulmonary embolism and deep vein thrombosis. Lancet.

[2] Essien EO, Rali P, Mathai SC (2019). Pulmonary embolism. Med Clin North Am.

[3] Rezende SM (2023). Barriers in the diagnosis of pulmonary embolism. Lancet Haematol.

[4] Giuntini C, Di Ricco G, Marini C, Melillo E, Palla A (1995). Pulmonary embolism: epidemiology. Chest.

[5] Blitzer RR, Eisenstein S (2021). Venous thromboembolism and pulmonary embolism: strategies for prevention and management. Surg Clin North Am.

[6] Li X, Haddadin I, McLennan G (2020). Inferior vena cava filter - comprehensive overview of current indications, techniques, complications and retrieval rates. Vasa.

[7] Choi JH, O'Malley TJ, Maynes EJ (2020). Surgical pulmonary embolectomy outcomes for acute pulmonary embolism. Ann Thorac Surg.

[8] Marti C, John G, Konstantinides S (2015). Systemic thrombolytic therapy for acute pulmonary embolism: a systematic review and meta-analysis. Eur Heart J.

[9] Rivera-Lebron B, McDaniel M, Ahrar K (2019). Diagnosis, treatment and follow up of acute pulmonary embolism: consensus practice from the PERT consortium. Clin Appl Thromb Hemost.

[10] Sheth SA, DeGeorge C, George A, Stead TS, Mangal R, Ganti L (2022). Deep venous thrombosis and pulmonary embolism secondary to mild traumatic injury in an elderly male with no additional risk factors. Cureus.

[11] Kim M, George A, Ganti L, Huang D, Carman M (2022). The burden of hypercoagulability in COVID-19. TH Open.

[12] Logan G, Dub L, Drone E, Ganti L, Webb AL (2020). Hypercoagulable state in COVID-19: a case series of three patients. Cureus.

